# FOXM1 contributes to taxane resistance by regulating UHRF1-controlled cancer cell stemness

**DOI:** 10.1038/s41419-018-0631-9

**Published:** 2018-05-11

**Authors:** Bowen Yuan, Youhong Liu, Xiaohui Yu, Linglong Yin, Yuchong Peng, Yingxue Gao, Qianling Zhu, Tuoyu Cao, Yinke Yang, Xuegong Fan, Xiong Li

**Affiliations:** 1 Center for Molecular Medicine, Xiangya Hospital, Central South University, Xiangya, China; 2 Hunan Key Laboratory of Molecular Radiation Oncology, Xiangya Hospital, Central South University, Xiangya, China; 3 Shenzhen BioScien Pharmaceutical Co. Ltd., Shenzhen, Guangdong China; 4 Hunan Key Laboratory of Viral Hepatitis, Xiangya Hospital, Central South University, Xiangya, China

## Abstract

Therapy-induced expansion of cancer stem cells (CSCs) has been identified as one of the most critical factors contributing to therapeutic resistance, but the mechanisms of this adaptation are not fully understood. UHRF1 is a key epigenetic regulator responsible for therapeutic resistance, and controls the self-renewal of stem cells. In the present study, taxane-resistant cancer cells were established and stem-like cancer cells were expanded. UHRF1 was overexpressed in the taxane-resistant cancer cells, which maintained CSC characteristics. UHRF1 depletion overcame taxane resistance in vitro and in vivo. Additionally, FOXM1 has been reported to play a role in therapeutic resistance and the self-renewal of CSCs. FOXM1 and UHRF1 are highly correlated in prostate cancer tissues and cells, FOXM1 regulates CSCs by regulating *uhrf1* gene transcription in an E2F-independent manner, and FOXM1 protein directly binds to the FKH motifs at the *uhrf1* gene promoter. This present study clarified a novel mechanism by which FOXM1 controls CSCs and taxane resistance through a UHRF1-mediated signaling pathway, and validated FOXM1 and UHRF1 as two potential therapeutic targets to overcome taxane resistance.

## Background

Taxane, including paclitaxel (Taxol), and docetaxel (Taxotere), has been widely used in cancer chemotherapy. Taxol has a significant role in the treatment of ovarian, breast, lung, head and neck, esophageal, prostate and bladder cancers, and Taxotere is effective in the treatment of breast, lung, head and neck, gastric, ovarian, and bladder cancers. Taxanes bind to β-tubulin, thereby reducing depolymerization. By stabilizing microtubules and dampening microtubule dynamics, taxanes prevent the formation of mitotic spindles, and chronically activate the spindle assembly checkpoint (SAC), which in turn leads to mitotic arrest and eventually induces cell death^1,2^. However, cancer cells develop resistance to taxanes. The molecular mechanisms by which cancer cells develop taxane resistance are not fully understood.

Taxane resistance is subclassified as innate resistance and acquired resistance. Acquired resistance results from the increased expression of drug efflux proteins such as ATP-binding cassette (ABC transporters)^3^, the altered expression and function of certain tubulin isotypes^4^, and the deregulation of Bcl-2 molecules^5,6^. Importantly, taxanes induced the expansion of stem-cell-like cancer cells, resulting in the development of taxane resistance and cancer relapse^7^.

FOXM1 is a cell proliferation-specific transcription factor that regulates the transcription of genes critical for the G1/S and G2/M cell cycle transition^8–10^. In addition to its roles as an oncogene^11^, FOXM1 overexpression is critical to the development of taxane resistance^12,13^. Several mechanisms have been reported for taxane resistance. FOXM1 increases drug efflux due to the upregulation of *abcc5* gene transcription^3^, promotes DNA damage repair through the transcriptional regulation of DNA repair genes^14^, drives abnormal mitotic spindle formation and mitotic catastrophe^5^ and upregulates apoptosis-associated molecules such as XIAP and Survivin^15^. In addition, FOXM1 regulates the stemness and self-renewal of cancer stem cells (CSCs) by directly regulating the gene transcription of CSC-associated genes^16^, or the crosstalk with CSC signaling pathways such as Wnt/β-Catenin^17,18^. The regulation of CSC expansion by FOXM1 is crucial for the development of taxane-resistance.

Compelling evidence suggests that the ubiquitin-like PHD and RING finger domain containing 1 (UHRF1), a key epigenetic regulator of DNA methylation, also contributes to the development of therapeutic resistance, including chemoresistance^19,20^ and radioresistance^21,22^. UHRF1 promotes DNA damage repair by regulating multiple DNA damage repair pathways, such as homologous recombination and the nonhomologous end joining (NHEJ) double-strand DNA repair pathway^23^. Additionally, UHRF1 controls the self-renewal and differentiation of stem cells^24^. Recent studies suggest that UHRF1 controls the self-renewal versus differentiation of hematopoietic stem cells by epigenetically regulating the cell-division modes^25^. Targeted deletion of *uhrf1* in epithelial basal stem cells results in premature cell senescence after injury without affecting cell survival or inducing premature differentiation^26^. However, no report is available about its functions in CSCs. RNA-seq data from recent studies indicated that UHRF1 might be regulated by FOXM1, and promoted the development of esophageal adenocarcinoma^27^. Whether FOXM1 regulates the maintenance and expansion of CSCs through a UHRF1-mediated signaling pathway is unknown.

In this study, we first established taxane-resistant cancer cells by long-term treatment with low doses of taxane. The stem-like cancer cells were expanded as taxane-resistant cancer cells. FOXM1 and UHRF1 were overexpressed in the taxane-resistant cancer cells, and positively regulated the maintenance of CSCs. FOXM1 and UHRF1 are also consistently expressed in prostate cancer tumor specimens and cells, with high correlation between the two molecules. Furthermore, we found that FOXM1 regulates CSCs and taxane resistance by directly regulating *uhrf1* gene transcription.

## Results

### Cancer cells developed taxane-resistance after long-term and intermittent exposure

**Fig. 1:**
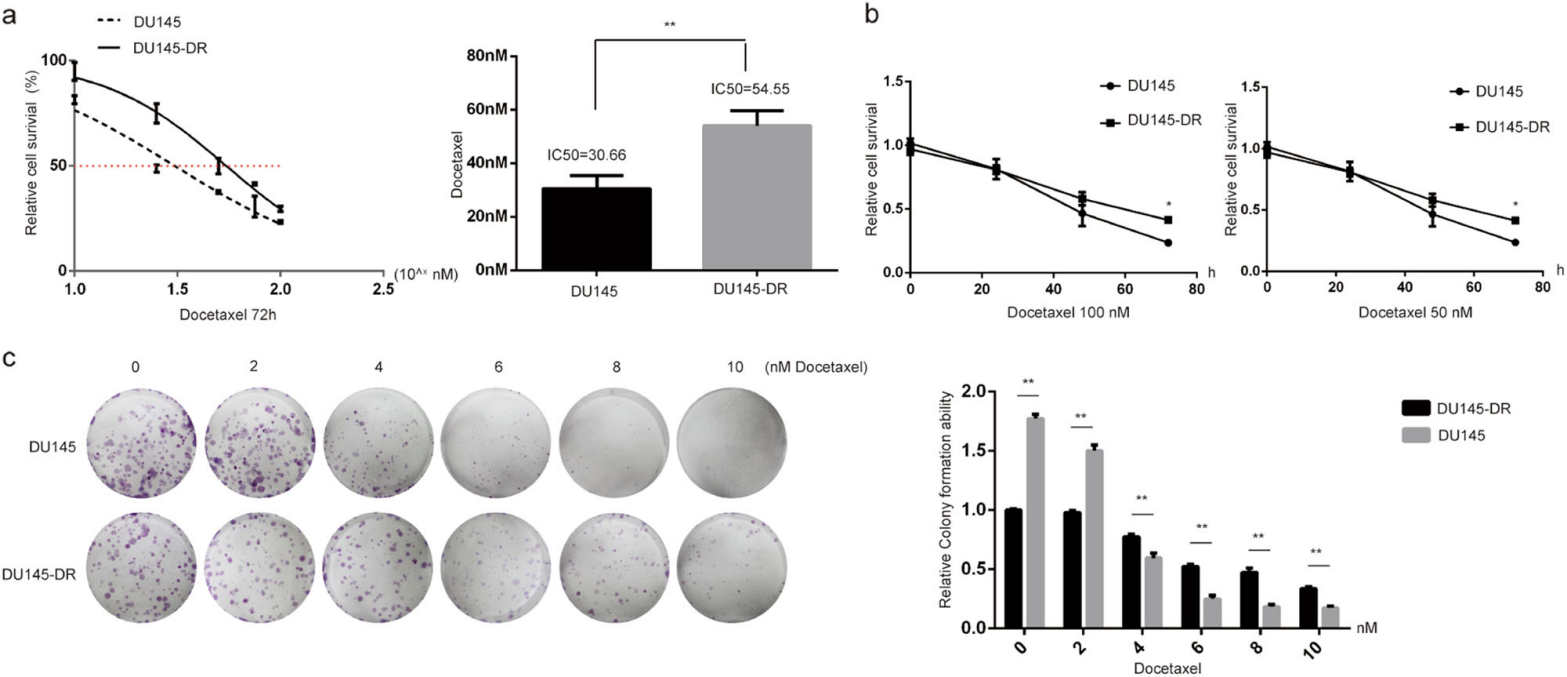
Assessment of drug resistance of DU145 and docetaxel-resistant DU145-DR cells. Docetaxel-resistant DU145 cells (DU145-DR) were induced. **a** The IC50 values of docetaxel were compared between DU145 and DU145-DR cells. The cells were treated with docetaxel at the doses shown. MTS assays were used to test cell viability 72 h after treatment. After three repeats for each dose, the relative cell survival was calculated (treatment vs. control) to compare the IC50 values. **b** Cell response to different doses of docetaxel with time. DU145 and DU145-DR cells were treated with paclitaxel at 50 or 100 nM individually, and cell viability was tested by a MTS assay 24, 48, and 72 h after treatment. The relative cell viability represents a ratio of docetaxel vs. control. **c** Cell colony formation assay. DU145 and DU145-DR cells were treated with docetaxel at stepwise concentrations for 48 h. One thousand cells were re-seeded in 6-well plates, and the cell clones formed were stained with crystal violet. The number of colonies was counted and the relative colony formation ability was analyzed 21 days after cell seeding

We previously developed a paclitaxel-resistant cell line, CNE2TR, by intermittently exposing CNE2 cells to low doses of paclitaxel over a long period^3,28^. In this study, we developed another docetaxel-resistant DU145 prostate cancer cell line (DU145-DR) using similar methods. We compared the drug sensitivity of DU145-DR cells to parental DU145 cells. The IC50 values of docetaxel in DU145-DR cells were significantly higher than DU145 (54.55 vs. 30.66 nM) (Fig. 1a). We tested the response of DU145 and DU145-DR cells to different doses of docetaxel (50 or 100 nM) over time. Cell viability significantly decreased with time. Comparatively, docetaxel killed more DU145 cells than DU145-DR cells 72 h after treatments (Fig. 1b). With docetaxel treatment in stepwise concentrations as shown in Fig. 1c for 48 h, more DU145-DR cell colonies formed than parental DU145 cells three weeks later. DU145-DR cells demonstrated much stronger resistance to docetaxel-induced cell killing (Fig. 1c).

### Docetaxel-resistant cancer cells acquired CSC characteristics

**Fig. 2:**
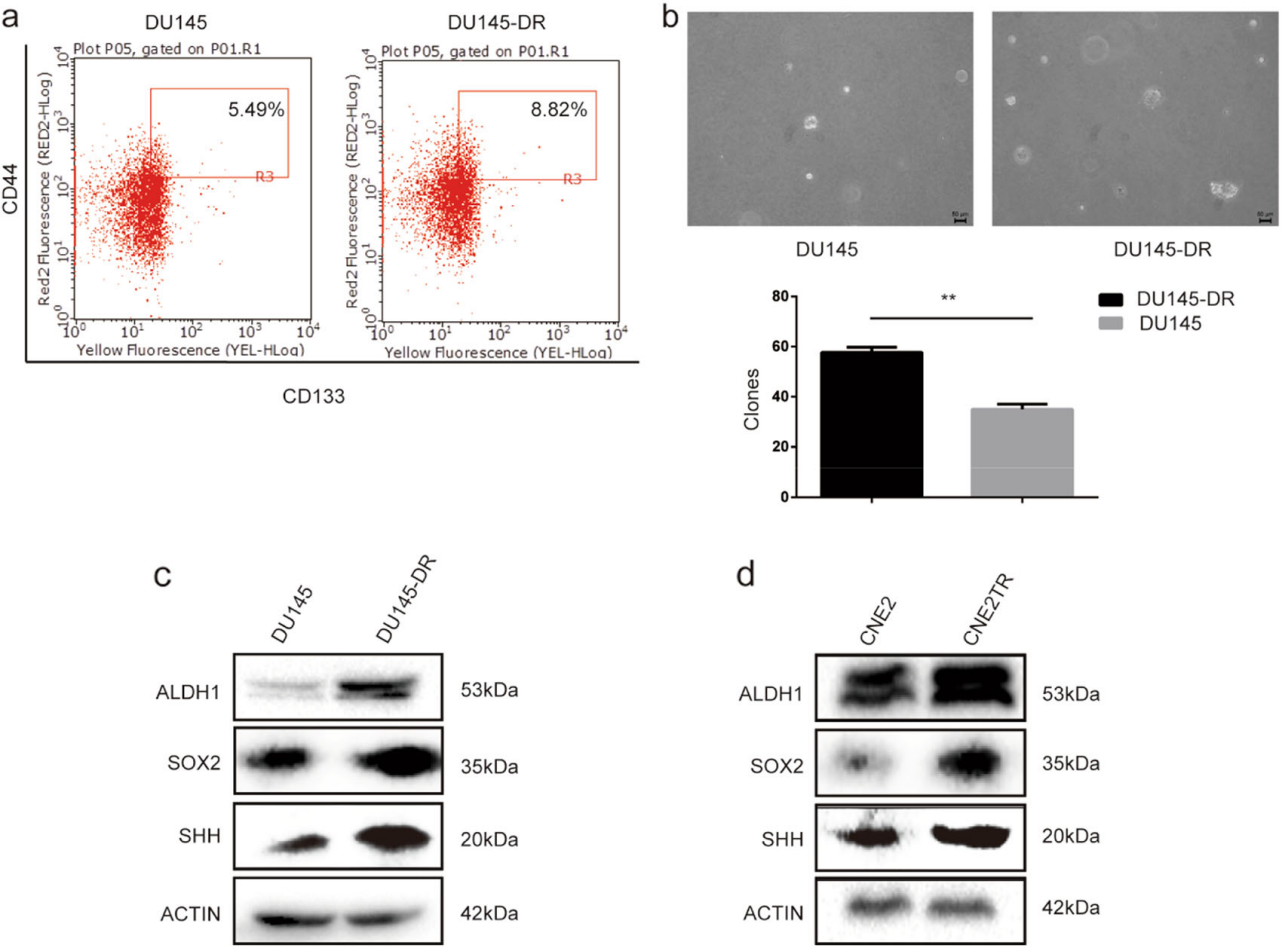
Docetaxel-resistant cancer cells acquired cancer stem cell (CSC) characteristics. **a** The CSC sub-population. DU145 and DU145-DR cells were labeled with fluorescent antibodies against CD44 (APC) and CD133 (PE). CD44^high^CD133^high^ cells were detected by flow cytometry. **b** The same number of DU145 and DU145-DR cells were plated in soft agar, and cell sphere formation was observed 21 days after cell seeding. The spheres were counted in five randomly selected fields, and the difference of spheres in two groups was compared by statistical analysis. **c** The protein levels of CSC-associated molecules such as ALDH1, SOX2, and SHH were tested in DU145 and DU145-DR cells by Western blot. **d** The protein levels of ALDH1, SOX2, and SHH were tested in CNE2 and CNE2TR cells by Western blot

We first tested the proportion of CSCs among DU145-DR and DU145 cell populations. The percentage of CD44^high^CD133^high^ cells in the DU145-DR population markedly increased compared to DU145 cells (8.82 vs. 5.49%, Fig. 2a). Cell spheres formed by DU145 cells were fewer and smaller than those formed by DU145-DR cells (Fig. 2b), and the protein levels of ALDH1, SOX2, and Sonic Hedgehog (SHH), typical stem cell markers in DU145-DR cells, were much higher than in the parental DU145 cells (Fig. 2c), indicating that the subgroup of docetaxel-resistant DU145-DR cells had acquired CSC characteristics. We also observed elevated protein levels of ALDH1, SOX2, and SHH in paclitaxel-resistant CNE2TR cells compared to CNE2 cells (Fig. 2d). These data indicated that the acquisition of CSC characteristics by a subpopulation of cancer cells contributes to taxane resistance.

### UHRF1 is overexpressed in taxane-resistant cancer cells, which contributes to maintenance of the CSC phenotype

**Fig. 3:**
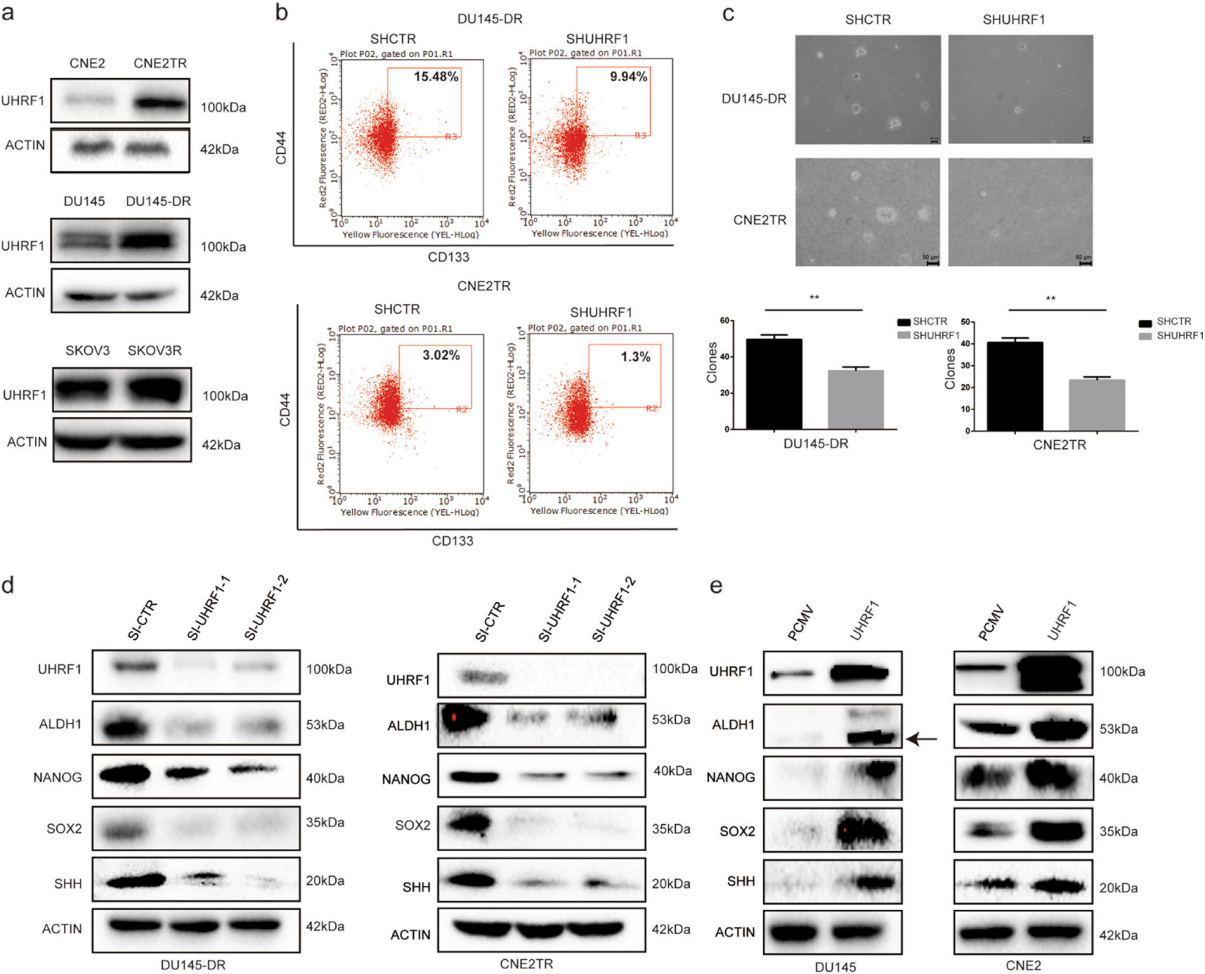
UHRF1 was overexpressed in the docetaxel-resistant cancer cells, which contributed to the maintenance of the CSC phenotype. **a** The protein levels of UHRF1 were analyzed in CNE2 and CNE2TR cells, DU145 and DU145-DR cells, and SKOV3 and SKOV3R cells by Western blot. **b** The depletion of UHRF1 in DU145-DR and CNE2TR cells decreased the CSC sub-population. DU145-DR and CNE2TR cells were infected with lentivirus-delivered shRNA-UHRF1 or shRNA-CTR. Cells were labeled with fluorescent antibodies against CD44 (APC) and CD133 (PE). CD44^high^CD133^high^ cells were detected by flow cytometry. **c** CNE2TR and DU145-DR cells were infected with lentivirus-delivered shRNA-UHRF1 or shRNA-CTR. One thousand cells were re-seeded in culture media-mixed agarose, and the cell spheres formed were stained with crystal violet and analyzed 21 days after cell seeding. The spheres were counted in five randomly selected fields, and the difference of spheres in two groups was compared by statistical analysis. **d** UHRF1 depletion decreased the expression of CSC-associated molecules ALDH1, NANOG, SOX2, and SHH in CNE2TR and DU145-DR cells. **e** UHRF1 overexpression elevated the levels of CSC-associated molecules ALDH1, NANOG, SOX2 and SHH in CNE2, and DU145 cells

UHRF1 has reported roles in the maintenance of both self-renewal and differentiation of hematopoietic stem cells^25^. We compared the protein levels of UHRF1 in CNE2 and CNE2TR cells, DU145 and DU145-DR cells, and SKOV3 and SKOV3R cells. Consistently in three pairs of cell lines, UHRF1 protein levels were significantly higher in docetaxel-resistant cells than the parental cancer cells (Fig. 3a). To validate the roles of UHRF1 in the regulation of CSCs, we depleted UHRF1 in DU145-DR and CNE2TR cells, and assessed the sub-population of CD44^high^CD133^high^ cells. The depletion of UHRF1 significantly decreased the CD44^high^CD133^high^ cell sub-population (Fig. 3b). UHRF1 depletion consistently and significantly decreased sphere formation by the two taxane-resistant cancer cell lines compared to the parental cancer cells (Fig. 3c). We also tested the protein levels of CSC-associated molecules ALDH1, NANOG, SOX2, and SHH with the depletion of UHRF1. Consistently in the DU145-DR and CNE2TR docetaxel-resistant cancer cells, protein levels of CSC-associated molecules declined with UHRF1 knockdown (Fig. 3d). To validate UHRF1 regulation of CSC-associated molecules, we tested protein levels when UHRF1 was overexpressed in the parental DU145 or CNE2 cells by transient transfection. The overexpression of UHRF1 significantly elevated the levels of CSC-associated molecules (Fig. 3e). These data identified the roles of UHRF1 in the maintenance of CSC characteristics.

### UHRF1 depletion re-sensitized cancer cells to docetaxel

**Fig. 4:**
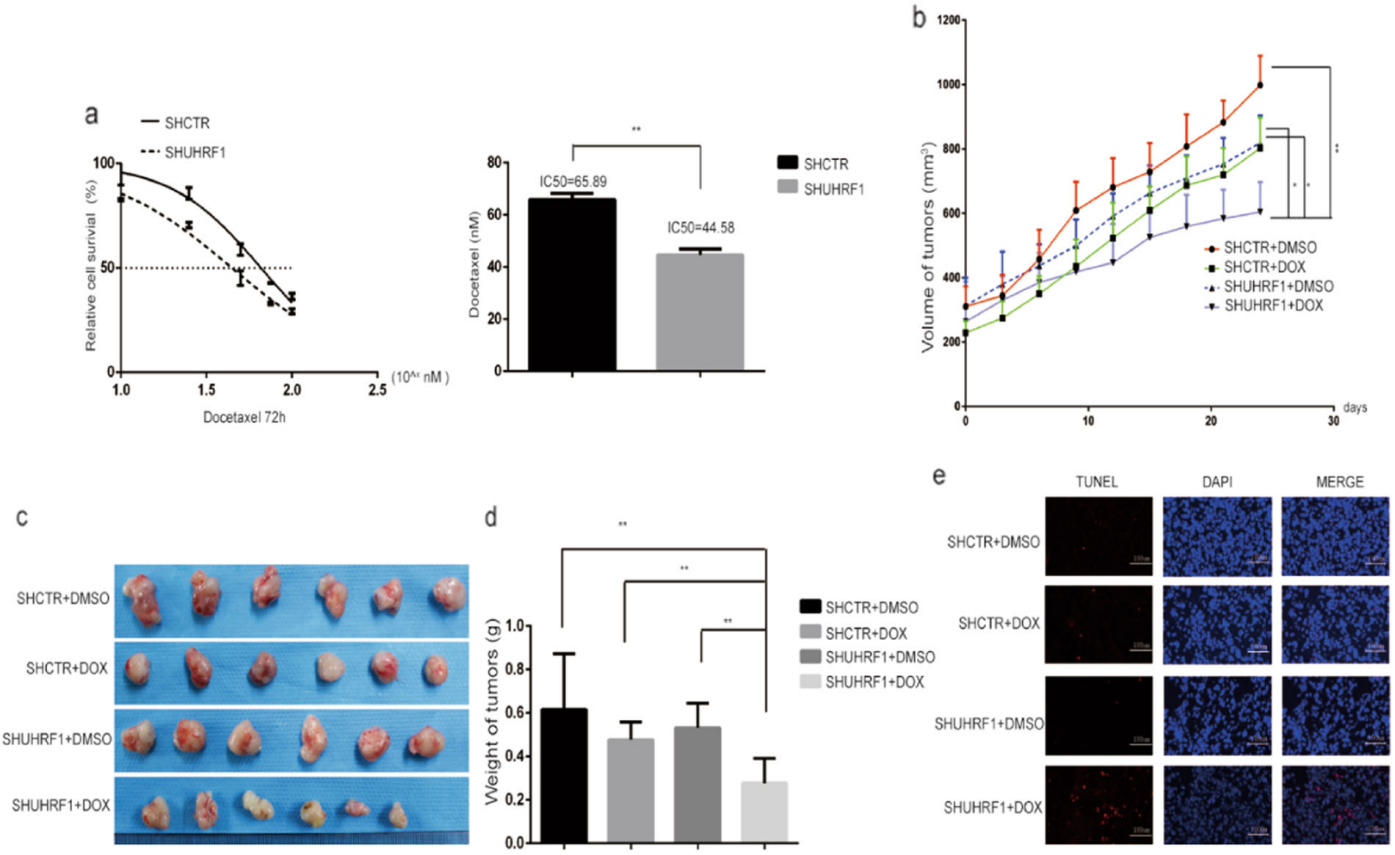
UHRF1 depletion re-sensitized cancer cells to docetaxel. **a** DU145-DR cells were infected with lentivirus-delivered shRNA-UHRF1 or shRNA-CTR, followed by puromycin drug selection. The IC50 values of docetaxel were compared between DU145-DR cells expressing the control shRNA and sh-UHRF1. **b** Tumor xenografts were established in immune-deficient nude mice with DU145-DR or UHRF1-depleted DU145-DR cells. Nude mice bearing tumor xenografts were treated with or without docetaxel (20 mg/kg) by i.p. injection once a week. The sizes of tumor xenografts were measured every 3 days. **c** The tumor masses were harvested from immune-deficient nude mice at the experimental endpoint. **d** The weight of tumors in each group was averaged and compared. **e** The tumor masses were immersed in embedding reagent OCT and frozen in liquid nitrogen. The frozen tissues were sectioned and stained with a One Step TUNEL Apoptosis Assay Kit. The nuclei were stained with DAPI. The TUNEL-positive red fluorescent cells were observed by fluorescent microscopy with 550 nm excitation and 570 nm emission wavelengths

Since UHRF1 plays a critical role in docetaxel resistance and maintenance of CSC characteristics, we depleted UHRF1 in DU145-DR cells by infecting cells with lentivirus-delivered shRNA-UHRF1 or shRNA-CTR. DU145-DR cells with UHRF1 stable depletion were selected by puromycin. Compared to DU145-DR cells expressing the control shRNA, the cells expressing sh-UHRF1 were more sensitive to docetaxel-induced cell killing (Fig. 4a, IC50 65.89 vs. 44.58 nM).

To confirm the in vitro results, we tested whether UHRF1 depletion reversed docetaxel resistance in vivo. Tumor xenografts were established in immune-deficient nude mice by inoculating DU145-DR or UHRF1-depleted DU145-DR cells. The nude mice bearing tumor xenografts were treated with or without docetaxel (20 mg/kg) by i.p. injection. The sizes of tumor xenografts containing UHRF1-depleted DU145-DR cells and treated with docetaxel were much smaller than those containing UHRF1-depleted DU145-DR cells or treated with docetaxel alone (Fig. 4b). The tumor masses were harvested at the endpoint of the experiment, and tumor size and weight were compared. The tumor size and average weight of tumors treated with UHRF1 depletion and docetaxel were much less than with the single treatment (Fig. 4c, d). The harvested tumors were rapidly frozen and sectioned, and the apoptotic cells inside the tumors were labeled with TUNEL staining. There were many more TUNEL-positive apoptotic cells in the double treatment group than with single treatment (Fig. 4e). The results showed that UHRF1 depletion significantly re-sensitized tumor xenografts containing docetaxel-resistant cells to docetaxel treatment.

### FOXM1 is overexpressed in docetaxel-resistant cancer cells, contributing to the maintenance of the CSC phenotype

**Fig. 5:**
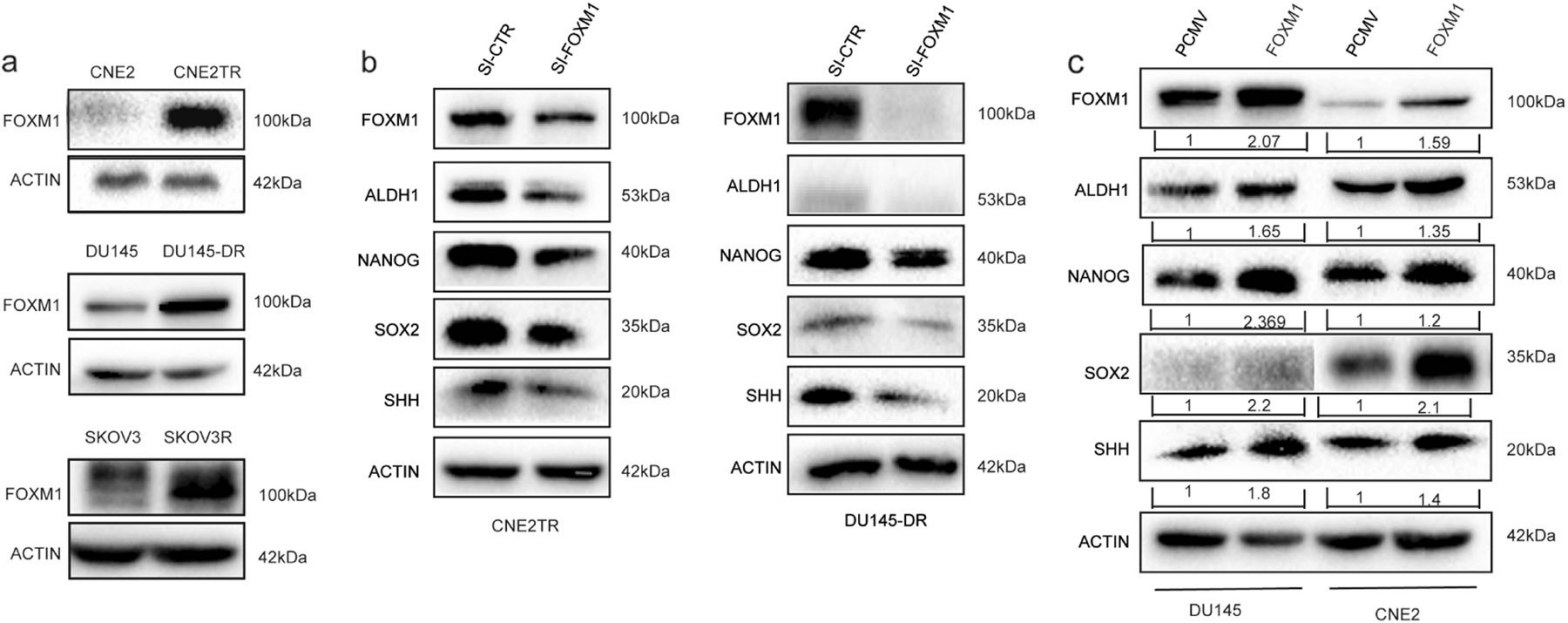
FOXM1 was overexpressed in the docetaxel-resistant cancer cells, which contributed to the maintenance of the CSC phenotype. **a** The protein levels of FOXM1 were analyzed in CNE2 and CNE2TR cells, DU145 and DU145-DR cells, and SKOV3 and SKOV3R cells by Western blot. **b** FOXM1 depletion decreased the expression of CSC-associated molecules ALDH1, NANOG, SOX2 and SHH in CNE2TR, and DU145-DR cells. **c** The ectopic overexpression of FOXM1 in DU145 or CNE2 cells elevated the expression levels of CSC-associated molecules ALDH1, NANOG, SOX2, and SHH

FOXM1 has been reported to affect drug resistance, including docetaxel resistance^29,30^. Additionally, FOXM1 contributes to drug resistance through the maintenance of CSCs^18,31^. We first compared FOXM1 protein levels between the docetaxel-resistant and parental cancer cells. Consistently in three pairs of cell lines, FOXM1 protein levels were significantly higher in docetaxel-resistant cells than in parental cancer cells (Fig. 5a). To validate the roles of FOXM1 in the regulation of CSCs, we depleted FOXM1 in docetaxel-resistant CNE2TR and DU145-DR cells, and assessed the protein levels of CSC-associated molecules ALDH1, NANOG, SOX2, and SHH. Consistently in the taxane-resistant DU145-DR and CNE2TR cancer cells, the protein levels of CSC-associated molecules declined with the knockdown of FOXM1 (Fig. 5b). Conversely, we tested the protein levels of CSC-associated molecules when FOXM1 was ectopically overexpressed in DU145 and CNE2 cells by transient transfection. As shown in Fig. 5c, the protein levels of CSC-associated molecules were elevated after ectopic overexpression of FOXM1. These data validated the critical roles of FOXM1 in the maintenance of the CSC phenotype.

### FOXM1 and UHRF1 were consistently expressed in prostate cancer tumor tissues and cell lines, and FOXM1 regulates UHRF1 expression

**Fig. 6:**
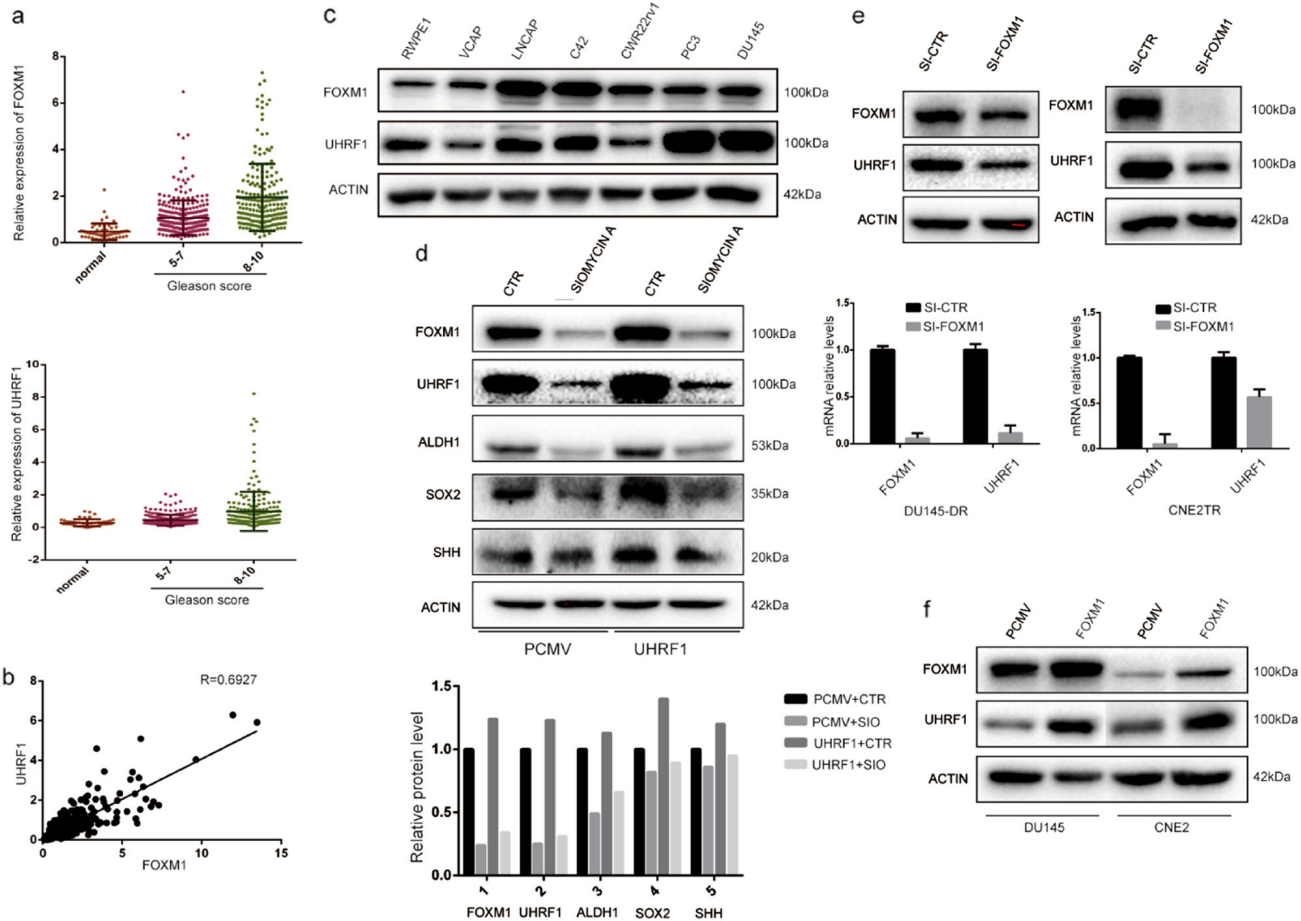
FOXM1 and UHRF1 are consistently expressed in prostate cancer tumor tissues and cell lines and FOXM1 regulates UHRF1 expression. FOXM1 and UHRF1 protein expression levels were analyzed in 546 prostate cancer tumor tissues using the TCGA data. The prostate cancer tumors were subclassified in three grades by Gleason score (low: 1–4, middle: 5–7, and high 8–10). **a** FOXM1 and UHRF1 expression levels were up-regulated as Gleason scores increased. **b** FOXM1 and UHRF1 protein levels showed a strong positive correlation in prostate cancer tissues (*R* = 0.6927). **c** FOXM1 and UHRF1 expression showed a high correlation in prostate cancer cells and normal prostate epithelial cells. **d** FOXM1 maintains CSC characteristics by regulating UHRF1. FOXM1 was depleted with a small molecule inhibitor, siomycin A, and UHRF1 was elevated by transient transfection. The expression levels of CSC-associated molecules ALDH1, SOX2, and SHH were tested by Western blot. **e** The depletion of FOXM1 with siRNA in CNE2TR and DU145-DR cells decreased the mRNA and protein levels of UHRF1. **f** The upregulation of FOXM1 in CNE2 and DU145 cells elevated the UHRF1 levels

We analyzed FOXM1 and UHRF1 protein expression levels in 546 prostate tumor tissues using the TCGA data. The prostate cancer tumors were classified in three grades by Gleason score. The patients in Gleason 1–4 were identified as low grade, 5–7 as middle grade, and 8–10 as high grade. The results showed that FOXM1 and UHRF1 expression levels increased along with the Gleason scores (Fig. 6a). FOXM1 and UHRF1 protein levels showed a strong positive correlation in prostate cancer tumor tissues (Fig. 6b, *R* = 0.6927). Additionally, we assessed the expression of FOXM1 and UHRF1 proteins in a panel of prostate cancer cell lines and non-malignant prostate epithelial cells. FOXM1 or UHRF1 expression levels showed high correlation in prostate cancer cells and non-malignant prostate epithelial cells (Fig. 6c).

Since both FOXM1 and UHRF1 regulate the CSC phenotype, we asked whether FOXM1 regulates the CSC phenotype through UHRF1-associated signaling pathways. We treated DU145-DR cells with a small molecule inhibitor of FOXM1 siomycin A. Consistent with FOXM1 siRNA, siomycin A significantly decreased the protein levels of CSC-associated molecules ALDH1, SOX2 and SHH, and the ectopic overexpression of UHRF1 reversed the siomycin A-induced decreases in CSC-associated molecules (Fig. 6d). The results showed that FOXM1 regulates the CSC phenotype through UHRF1. In DU145-DR and CNE2TR cells, we depleted FOXM1 with siRNA, and tested the protein expression of UHRF1. The depletion of FOXM1 significantly decreased the mRNA and protein levels of UHRF1 (Fig. 6e). Conversely, we tested the protein levels of UHRF1 in DU145 and CNE2 cells when FOXM1 was ectopically overexpressed by transient transfection. FOXM1 elevation increased the expression level of UHRF1 (Fig. 6f). The data suggested that FOXM1 regulates the gene expression of UHRF1.

### FOXM1 regulates *uhrf1* gene transcription by directly binding to the *uhrf1* gene promoter

**Fig. 7:**
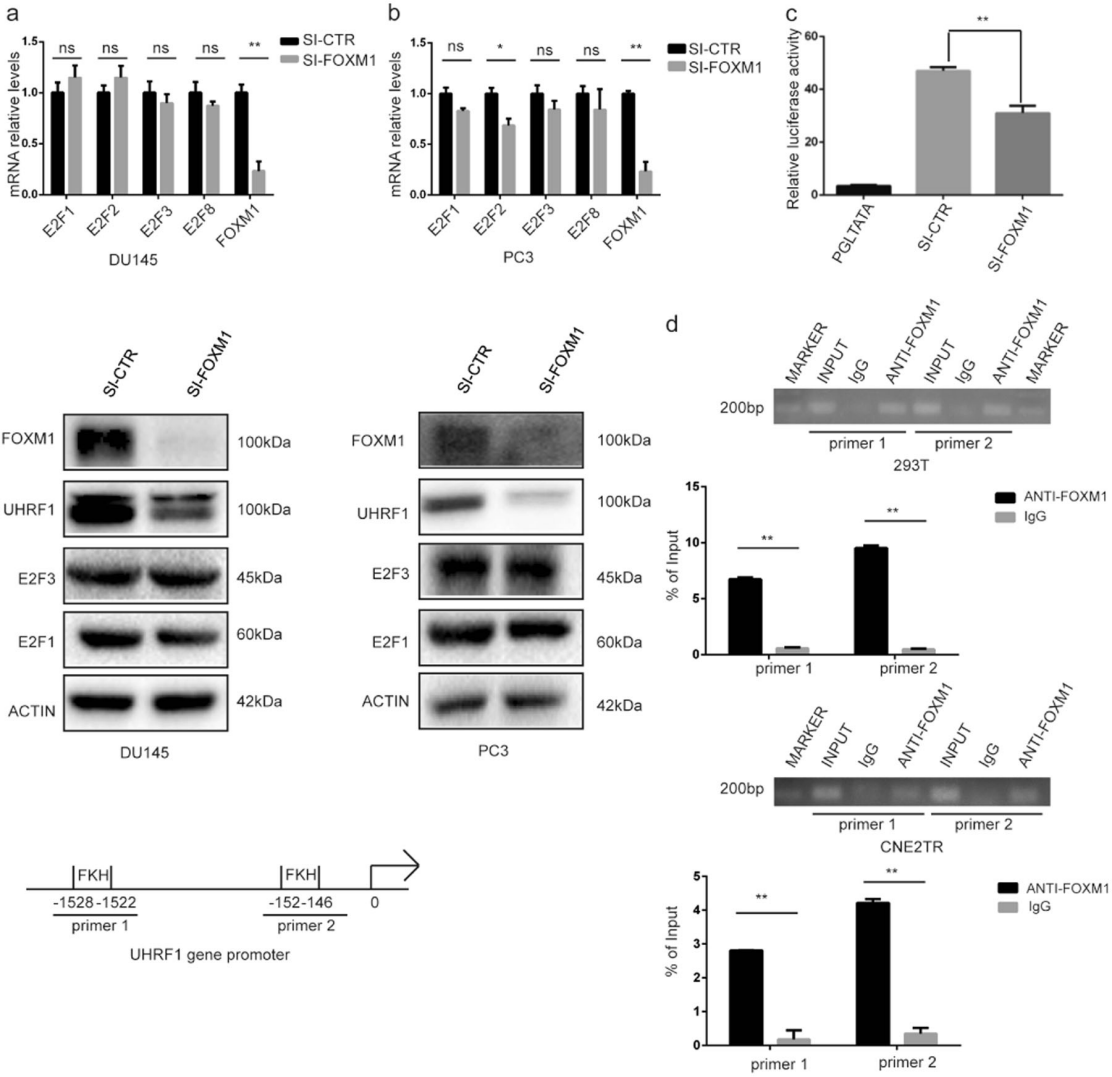
FOXM1 regulates *uhrf1* gene transcription. **a** FOXM1 regulates *uhrf1* gene transcription, but not through the E2F pathway. FOXM1 was depleted with siRNA in DU145 cells, and the mRNA levels of *uhrf1, e2f1, e2f2, e2f3,* and *e2f8* genes were tested by RT-PCR. The protein expression of E2F1 and E2F3 was tested when FOXM1 was depleted with siRNA. **b** FOXM1 was depleted with siRNA in PC-3 cells, and the mRNA levels of *uhrf1, e2f1, e2f2, e2f3,* and *e2f8* genes were tested by RT-PCR. The protein expression of E2F1 and E2F3 was tested when FOXM1 was depleted with siRNA. **c** The depletion of FOXM1 with siRNA in HEK-293T cells decreased *uhrf1* gene promoter activity. **d** FOXM1 directly binds to *uhrf1* gene promoter. The FOXM1 protein-bound DNA in HEK-293T and CNE2TR cells was purified by ChIP, and primers spanning the FKH binding motif at the *uhrf1* gene promoter were designed. FOXM1 protein binding to the *uhrf1* gene promoter was detected by ChIP-PCR

The gene transcription of *uhrf1* is reported to be regulated by E2F molecules^32^. To clarify whether FOXM1 regulates *uhrf1* gene transcription through the E2F pathway, we tested the expression of *E2F1*, *E2F2*, *E2F3*, and *E2F8* when FOXM1 was depleted in DU145 and PC3 cells with siRNA. We did not observe a clear impact on E2F molecules at the mRNA and protein levels (Fig. 7a,b). We further tested the impact of FOXM1 on the promoter activity of *uhrf1* gene. A plasmid using a 2000bp fragment of *uhrf1* gene promoter controlling the luciferase gene was constructed. We tested the impact of FOXM1 on *uhrf1* gene promoter activity in HEK-293 cells when FOXM1 was depleted with siRNA. The depletion of FOXM1 significantly decreased the *uhrf1* gene promoter activity (Fig. 7c). We further tested the DNA binding of FOXM1 to *uhrf1* gene promoter by ChIP-qPCR. The forkhead box (FKH) consensus motif was identified in the *uhrf1* gene promoter, and the FOXM1 protein-bound DNA in HEK-293 and CNE2TR cells was purified by ChIP, and specific PCR primers spanning the FKH binding motif were designed. Consistently, FOXM1 directly bound to the *uhrf1* gene promoter (Fig. 7d). These data verified that FOXM1 regulates *uhrf1* gene transcription by direct DNA binding to the FKH motif at the *uhrf1* gene promoter.

## Discussion

The expansion of CSCs after treatment has been identified as one of the most important factors responsible for acquired therapeutic resistance. To study the association of taxane resistance and CSCs, we generated taxane-resistant cancer cells by treating cells with a high dose of taxane, and maintaining low dose treatment over a long period (Fig. 1). The subpopulation of CSCs significantly increased along with the establishment of acquired taxane resistance, and CSC-associated molecules such as ALDH1, SOX2, and SHH were remarkably elevated (Fig. 2). These results showed that the expansion of the CSC subpopulation is indeed a critical factor in the development of taxane resistance.

FOXM1 and UHRF1 both have reported roles in therapeutic resistance, including taxane resistance, as well as in the regulation of stem cell self-renewal and differentiation ^5,13,17,19,21^. In addition, FOXM1 and UHRF1 are highly correlated in tumor specimens and prostate cancer cell lines (Fig. 6). In our previous studies, we found that FOXM1 is overexpressed in paclitaxel-resistant cancer cells, and FOXM1 depletion overcame the paclitaxel-resistance by decreasing drug efflux^3^. In this study, FOXM1 is closely associated with the stemness of taxane-resistant cancer cells. FOXM1 is overexpressed in taxane-resistant cancer cells, and the depletion or elevation of FOXM1 accordingly changed the expression levels of CSC-associated molecules (Fig. 5). These results showed that FOXM1-regulated CSC stemness was responsible for the taxane-resistance. However, the roles of UHRF1 in the expansion of CSCs in taxane-resistance are not well known. In this study, UHRF1 overexpression was detected in taxane-resistant cancer cells, and the depletion of UHRF1 correspondingly lowered the expression of CSC-associated molecules and decreased the subpopulation of CSCs and sphere formation ability. Furthermore, UHRF1 depletion significantly promoted sensitivity to docetaxel in a prostate cancer xenograft model (Fig. 4). This is the first report that UHRF1 plays a critical role in the expansion of CSCs and development of acquired therapeutic resistance.

Gene transcription of *uhrf1* is regulated by several identified transcription factors. E2F1 and E2F8 are two well-known transcription factors that control UHRF1 expression^33,34^. Specificity protein 1 (SP1) is a transcription factor directly binding to *uhrf1* gene promoter, and 3,3′,5-Triiodo-L-thyronine (T3)/thyroid receptor (TR) downregulated UHRF1 in HepG2 cells by repressing SP1 binding^35^. Transcription factor Yingyang 1 (YY1) is a mediator for G9a recruitment binding to the *uhrf1* gene promoter, and represses *uhrf1* gene transcription in the H1299 lung cancer cell line^36^. A recent study indicated by ChIP-seq that UHRF1 may be a direct target of FOXM1 transcription factor in the esophageal adenocarcinoma-derived OE33 cell line^27^. However, the exact mechanism is elusive. Our present study addressed whether FOXM1 regulates taxane resistance and CSCs through a UHRF1-mediated signaling pathway. In our present report, the depletion or elevation of FOXM1 significantly influenced UHRF1 expression at both the mRNA and protein levels. Siomycin A, a small molecule inhibitor of FOXM1, decreased the expression of CSC-associated molecules, while UHRF1 overexpression reversed the decrease of CSC-associated molecules. The results suggested that FOXM1 regulates the stemness of CSCs through a UHRF1-mediated signaling pathway (Fig. 6). Our further studies clarified that FOXM1 regulates *uhrf1* gene transcription in an E2F-independent manner, and by directly binding to the FKH motif at the promoter.

Altogether, our results in this study identified UHRF1 as a critical regulator of CSCs and taxane resistance, and demonstrated that FOXM1 is an important transcription factor regulating both the stemness of CSCs and taxane resistance by regulating *uhrf1* gene transcription. FOXM1 and UHRF1 may both be therapeutic targets to overcome taxane resistance.

## Materials and methods

### Cell culture

Prostate cancer cell lines PC3 and DU145, and ovarian cancer cell line SKOV3 cells were purchased from ATCC (Manassas, MA, USA). Nasopharyngeal cancer (NPC) cell line CNE2 was purchased from the Cancer Research Institute of Central South University (Changsha, Hunan, China). The paclitaxel resistant cell line CNE2TR were generated by the methods as described in a previous publication^37^. The docetaxel resistant cell line DU145-DR was generated by treating cells with high doses of docetaxel (50 nM), and maintaining the residual colonies at low doses (20 nM) over 12 weeks. SKOV3R cells were kindly gifted from Dr. Yu Zhang from Xiangya Hospital Central South University. These cells were cultured in RPMI-1640 media supplemented with 10% FBS and 1% streptomycin/penicillin.

### Plasmids, siRNA, and shRNA

FOXM1 and UHRF1 cDNAs in pCMV-XL5 vector were purchased from Origene (Rockville, MD, USA). A 2000 bp *uhrf1* gene promoter was obtained by the PCR method using genomic DNA as the template, and then subcloned to PCR2.1 vector (Thermo Fisher Scientific, Shanghai, China). pGL3-UHRF1-Luc was generated by cutting the *uhrf1* gene promoter fragment with *Kpn1* and *HindIII*, then inserting to the pGL3-basic vector. The sequences of siRNAs of FOXM1 and UHRF1 were as follows: the sense sequence of siRNA FOXM1: 5′-CUCUUCUCCCUCAGAUAUATT-3′, and the antisense sequence: 5′-UAUAUGAGGGAGAGTT-3′; the sense sequence of siRNA URHF1 #1: 5′-GCGCUGGCUCUCAACUGCU-3′, and the antisense sequence: AGCAGUUGAGCCAGCGC-3′; the sense sequence of siRNA UHRF1 #2: 5′-GCAUCUACAAGGUUGUGAA-3′, and the antisense sequence: 5′-UUCACAACCUUGUAGAUGC-3′. The primers 5′-CCGG–GCGCUGGCUCUCAACUGCU-CTCGAG-AGCAGTTGAGAGCCAGCGC-TTTTT-3′ were designed to synthesize UHRF1shRNA.

### Transient transfection

cDNA transient transfection was conducted using lipofectamin 2000 (Thermo Fisher Scientific, Wilmington, DE, USA). siRNA transfection was conducted using DharmaFECT Transfection Reagent (Thermo Fisher Scientific). The experimental protocol was modified from the manufacturer’s manuals.

### Antibodies and chemicals

The primary antibodies anti-FOXM1, anti-UHRF1, anti-ALDH1, anti-NANOG, anti-E2F1, anti-E2F3, anti-β-ACTIN were purchased from Santa Cruz Biotechnology (Dallas, UT, USA). Anti-SHH antibody was purchased from Cell Signal Transduction (CST, Danvers, MA, USA) and anti-SOX2 was purchased from ABclonal (Woburn, MA, USA). The small molecule inhibitor of FOXM1 Siomycin A was purchased from Sigma Aldrich (St. Louis, MO, USA) and Docetaxel was purchased from Selleck Chemicals (Houston, Texas, USA).

### RT-PCR

The total RNA was extracted from cells using a RNAiso Plus kit (Takara Bio Inc, Shiga, Japan). The concentration of RNA was measured by spectrophotometer and the RNA was reverse transcripted to cDNA (Takara Bio Inc). Quantitative real-time PCR was performed using the Applied Biosystems 7500 Real Time PCR system (Thermo Fisher Scientific).

### Flow cytometric analysis

Cells suspended with PBS were inoculated with anti-CD133 (PE-conjugated, MiltenyiBiotec, San Diego, CA, USA) and anti-CD44 (APC-conjugated, BD PharMingen, San Jose, CA, USA) at 37 °C for 20 min. The positive cells were analyzed by flow cytometry (Millipore, Temecula, CA, USA).

### Luciferase reporter assay

HEK-293T cells were transfected with pGL3-UHRF1-Luc, together with siRNA-FOXM1 or siRNA-CTR (control) using the TransIT-X2 Dynamic Delivery System (Mirus, Madison, WI, USA), with pRL-SV40 as a transfection efficiency control. The cells were frozen/thawed for two cycles in lysis buffer (Genecopoeia, Rockville, MD, USA), and firefly/Renilla luciferase activities were tested by luminometer.

### Cell proliferation assay (MTS)

The cells were plated on 96-well plates (5000 cells per well) and treated with 50 or 100 nM docetaxel. The cells were stained with MTS solution [3-(4, 5-dimethylthiazol-2-yl)-5-(3-carboxymethoxyphenyl)-2-(4-sulfophenyl)-2H-tetrazolium] (Promega, Madison, WI, USA) at 37 °C in 5% CO_2_ for 1 h. Cell viability was assessed by measuring the 490 nM absorbance in a spectrometer.

### Colony formation assay

DU145 and DU145-DR cells were seeded in 6-well plates (500 cells per well) and exposed to docetaxel at different concentrations (0, 2, 4, 6, 8, 10 nM) for 48 h. Docetaxel was washed away and then the cells were maintained for another 2 weeks for colony formation. The cell colonies were fixed with 3.7% paraformaldehyde and stained with 1% crystal violet solution. The cell colonies were dissolved in 1% SDS, and cell survival was evaluated by measuring the absorbance at 570 nM.

### Chromatin immunoprecipitation (ChIP)-PCR

HEK-293T or CNE2TR cells were plated in 100 mm culture dishes. The cells were cross-linked with 1% paraformaldehyde when cell confluence attained nearly 90%. The cells were lysed and the DNA fragment was sonicated to shear a mean DNA fragment size of about 500 bp. The chromatin–protein complex was incubated with FOXM1 antibodies on rotating platform overnight at 4 °C, with IgG as the negative control. Five percent of the total lysate was used for input control. The immune complexes were harvested with beads, and then de-crosslinked and the chromatin DNA fragments purified. The degree of FOXM binding to chromatin DNA was analyzed by semi-quantitative PCR and quantitative RT-PCR. The PCR primers spanning the FKH binding motif at the uhrf1 gene promoter were designed as follows: Primer 1 forward: AAAGACAGCAAACAAGCCCTG, and reverse: CTCGCACGCATTGACCAGTA; Primer 2 forward: CACTTGGTTGAGTTCCCCCG, reverse: GAAGGTCCAACCCATCCCTC. The binding efficiency of FOXM1 was calculated by following the calculation formula described in a previous publication^38^.

### Animal experiments

The animal experiment protocol was approved by the Ethics Committee of Xiangya Hospital Central South University. DU145-DR or UHRF1-depleted DU145-DR cells were injected (5 × 10^6^/100 μL) into the subcutaneous tissue of the left flank region of 6-week-old immune-deficient nude mice (BALB/C-nu/nu, SLAC Laboratory, Shanghai, China). The nude mice bearing tumor xenografts were treated with or without docetaxel (20 mg/kg) by i.p. injection once a week. The sizes of tumor xenografts were measured every 3 days, and volume (*V*) was calculated using the following formula: *V* = *ab*^2^/2 (*a*: the long diameter and *b*: the short diameter). The mice were sacrificed when tumor volume reached 1000 mm^3^ or the 28 days after treatments. The tumor masses were harvested at the experiment endpoint, and the size and weight of tumors in each group was averaged and compared.

### TUNEL assay

The tumor masses were harvested from subcutaneous xenografts of nude mice at the experiment endpoint, and immediately immersed in OCT solution and frozen in liquid nitrogen. The frozen tissues were sectioned and stained using a One Step TUNEL Apoptosis Assay Kit (Beyotime, Shanghai, China) according to the manufacturer’s instruction. The nuclei were stained with DAPI. TUNEL-positive red fluorescent cells were observed by fluorescent microscopy with 550 nm excitation and 570 nm emission wavelengths.

### TCGA data analysis

For mRNA sequencing data, the gene level and exon level quantification in Fragmants Per Kilobase of transcript per Million mapped reads (FPKM) were generated by Genomic Data Commons (GDC). These data can be downloaded from the Cancer Genome Atlas website (https://cancergenome.nih.gov/). UHRF1 and FOXM1 levels were analyzed according to the prostate cancer Gleason scores, and the correlation analysis of FOXM1 and UHRF1 molecules was calculated using GraphPad 6.0 (GraphPad Software, Inc. La Jolla, CA, USA).

### Statistics

All in vitro experiments were done at least in triplicate. The data were presented as the mean ± SD. All statistical analysis was conducted with SPSS software. The statistical difference between two samples was analyzed by Students *t* test. The comparison of tumor sizes in four groups in the animal study was analyzed using one-way ANOVA. *P < 0.05; **P < 0.01 was considered statistically significant.
